# Can learning about trials be child’s play? A qualitative exploration of the ‘Schools Teaching Awareness of Randomised Trials’ (START) initiative

**DOI:** 10.1186/s13063-020-4130-9

**Published:** 2020-02-19

**Authors:** Linda Biesty, Sandra Galvin, Elaine Finucane, Patricia Healy, Declan Devane, Tom Conway

**Affiliations:** 1 School of Nursing and Midwifery, National University of Ireland Galway, Galway, Ireland; 2 Health Research Board - Trials Methodology Research Network, National University of Ireland Galway, Galway, Ireland; 3 Evidence Synthesis Ireland, University Road, Galway, Ireland; 4 Cochrane Ireland, Galway, Ireland; 5 Health Research Board - Clinical Research Facility Galway, National University of Ireland Galway, Galway, Ireland

**Keywords:** Trials, RCTs, Methodological research, Qualitative research

## Abstract

**Background:**

The Health Research Board-Trials Methodology Research Network (HRB-TMRN) celebrates International Clinical Trials Day with the help of the younger members of our community through the Network’s ‘Schools Teaching Awareness of Randomised Trials (START)’ initiative. START seeks to increase public awareness of randomised trials in Ireland. Launched in 2016, it asks children (8–12 years old) to conduct and report their very own fun randomised trial. The study reported in this paper sought to explore children and teachers perceptions and experiences of the START initiative.

**Methods:**

We conducted eight, one-to one interviews with teachers and eight focus groups with 61 children who took part in the 2018 START initiative. Interviews and focus groups were recorded and transcribed and the data analysed using template analysis.

**Results:**

The findings of this study highlight the benefits of participating in START and the areas of the initiative that required further attention. Teachers and children recalled the benefits of experiential learning associated with START and *learning by doing* encouraged a fun way of engaging with trial processes. By recalling all aspects of planning, conducting and reporting their trial, the children in this study demonstrated their awareness of the trial processes. The teachers suggested that START provides a valuable framework to contribute to key aspects of the primary school curriculum in Ireland. The experiences of these participants also provided recommendation for improving the programme for future START participants.

**Conclusions:**

Increasing public awareness and understanding of randomised trials can help increase public engagement in trials. By educating children about the importance of trials and supporting them to ‘learn by doing’ by carrying out their own trial, the START initiative can contribute substantially to children’s awareness and understanding of trial processes. Given that children are the public, the patients and the researchers of the future, initiatives such as START deserve attention.

## Background

The Health Research Board-Trials Methodology Research Network (HRB-TMRN, Ireland) celebrates International Clinical Trials Day annually on May the 20th with primary schools through the Network’s ‘Schools Teaching Awareness of Randomised Trials (START)’ initiative. START is an outreach initiative that incorporates both a competition for primary schools and an engaging day of interactive scientific workshops at the National University of Ireland Galway for the top three school entrants. Launched in 2016, START is a public engagement initiative that challenges school children (8–12 years old), with the guidance of their teachers, to design, conduct, analyse and report their very own randomised trial.

A strategic goal of the HRB-TMRN is to increase public awareness and understanding of randomised trials. This is done through several public engagement initiatives, of which START is one (www.thepeoplestrial.ie is another example). START focuses on children drawing on their natural inquisitiveness and their enthusiasm to engage with new learning experiences [1]. The Theory of Experiential Learning [2, 3] underpins START. This theory suggests that learning occurs when people are involved personally in the learning experience [4]. The purpose of the competition aspect of START encourages primary school students to become aware of the trial process and gain insights into the processes needed to conduct a fair comparison. While entrants are encouraged to focus on simple and fun trial questions, they are required to use the steps of the trial process to answer their question.

START is promoted via a dedicated website (www.STARTcompetition.com), Twitter (@STARTSchools) and the national media in Ireland. Information in relation to START has been posted and emailed to over 3125 primary schools in Ireland.

To date, 45 trials have been submitted to the START competition. Some questions posed include: Can using coloured paper for written spelling tests increase students’ scores compared to white paper? Can interactive spelling games improve spelling test results compared to teaching not using interactive games? Can having classes outside help students attention during class compared to having classes inside?

All classes and teachers can access to a step-by-step guide on the START website. This guide includes resources that were developed by trialists in consultation with a primary school principal (head teacher) specifically for START. These resources explain the trials process in a distilled manner for both teachers and students by providing prompts and simple guidance for each stage of a trial (www.STARTcompetition.com).

Anecdotally, START has been received well by primary school students and their teachers, by the research community and by the Health Research Board (as the funder) and has captured the imagination of the public. However, the children and teachers involved in this initiative have not had a formal platform to voice their perceptions and experiences of START. This study provided such an opportunity.

## Methods

The aim of this qualitative descriptive study was to explore the START initiative from the perspective and experiences of participating children and their teachers. Our objectives were to (a) explore the experiences of teachers and children of taking part in the initiative and (b) identify what they felt worked well and areas for improvement of the initiative.

### Methodology

We used a descriptive qualitative study, which we report in line with the Standards for Reporting Qualitative Research (SRQR) [5] (Additional file 1).

While qualitative research incorporates many philosophies and approaches [6, 7], all endeavour to gather information directly from those experiencing the phenomena under exploration [8, 9]. Such an approach to this work ensured that the children and teachers were given a formal platform to voice their perceptions and experiences of the START initiative.

The goal of qualitative descriptive research is to provide an overview of real life situations in the everyday language of the individuals involved [10, 11], enabling researchers to present the findings as “a rich, straight description of experiences, perceptions, or events” (p. 53) [12] using language from the collected data. This design allowed us to maintain the voice of the participants and to ensure that the findings remained grounded in their language and description.

### Sample

Participants were children and their teachers who took part in START 2018 and we used a purposeful sampling technique [13]. In line with good trial practices, schools are asked to register their trial protocol for the START competition before they commence their trial and to identify a teacher as a point of contact for their study. Eleven schools registered for START 2018 and eight schools indicated that they were willing to receive information about this qualitative study. Participant information packs (consisting of covering letters, participant information leaflets and consent forms for teachers, parents/guardians and children) were sent to these schools for the attention of the contact teacher. All teachers in receipt of information packs agreed to participate in this study (*n* = 8).

Teachers were asked to distribute the participant information packs to children participating in START 2018 and their parents/guardians. We sought written parental/guardian consent and the agreement/assent of the child themselves to take part in the focus group interview. For some of the focus group interviews, the class identified the children they wanted to represent their views; for other groups the teacher identified the children to first complete and return their consent forms. This stage of recruitment yielded a sample of 61 children.

The recruitment phase occurred before the schools knew their ranking in the START competition; the eight schools who took part provided a mix of groups that did and did not attend the workshop day at NUI Galway. The schools were located in urban and rural areas across Ireland; the smallest school had 60 students in total, the largest had 560 students. The children participating in this study were in 4th, 5th and 6th class (10–12 years old).

### Ethical issues

Ethical approval to conduct this study was provided by the Research Ethics Committee at the National University of Ireland Galway (reference number 18-Jan-01). The ethical principles guiding all studies applied to this research (e.g. informed consent, protection and safety of the participants, respect to the rights and wishes for those participating). There were also specific issues that we had to consider given that children formed part of the participant population. We were guided by the ‘Children First: National Guidance for the protection and Welfare of Children’ [14] and the ‘Guidance for developing ethical research projects involving children’ [15]. These guidelines identify best practice in relation to promoting child protection and identify a pathway of reporting should concerns in relation to the welfare of a child arise. This pathway was built into our study protocol. In line with the guidelines for conducting research with children we obtained written parental/guardian consent and also the agreement/assent of the child themselves prior to any data collection. Children could themselves read or have the information and consent forms read to them prior to providing assent. Written informed consent was also obtained from all teachers participating in this study.

Children participated in focus groups facilitated by two to three researchers. All the research team participated in the University’s Garda Vetting process in line with the National Vetting Bureau, Children and Vulnerable Person Act (2012).

### Data collection

Eight semi-structured, one-to-one interviews with teachers (*n* = 8) and eight semi-structured focus groups with children (*n* = 61) were conducted in May and June 2018. These interviews and focus groups took place in the individual schools at a time identified, by the teachers, as convenient. TC (an experienced qualitative researcher) conducted all the interviews with the teachers. TC led the focus groups with the children and other members of the team (LB, DD, EF and PH) moderated and took field notes**.** Conducting focus groups with the children ensured a larger number of children contributed to this study than would have been possible if we conducted individual interviews with each child during their school day. Their interactions during the focus groups also provided interesting insights that we may not have captured during one on one interviews.

The interviews and focus groups were conducted using two semi-structured interview guides adapted from those developed by the Informed Health Choices Initiative (https://www.informedhealthchoices.org/publications/). The IHC guides were used in a qualitative study conducted as part of the process evaluation of an intervention to teach primary school children to assess claims about treatment effects. These guides focused on gaining an understanding of teachers’ and children’s personal feelings, perceptions and opinions about the intervention [16]. We adapted the guides so questions and prompts focused on eliciting the teachers’ and children’s experiences of START and also what they felt worked well and areas for improvement.

All interviews and focus groups were recorded and transcribed verbatim. Interviews with teachers ranged in length from 22 to 44 min (average 32 min). The focus groups with the children ranged from 27 to 51 min (average 37 min). The largest focus group consisted of 12 children (at the request of the school) and the smallest had five participants (the average number of children in a focus group was eight). Field notes were recorded after the interviews (by the interviewer) and also during the focus groups by the researcher moderating. These notes were used to inform the interpretation of the findings, e.g. to highlight any emotion or nonverbal communication that may have occurred during data collection.

### Data analysis

All interviews and focus groups were analysed using template analysis as described by King and Brooks [17–19]. Template analysis, a form of thematic analysis, provided a way to explore and combine the data from two different participant groups in one cohesive report. It also allowed for two members of the research team to analyse the data (TC and LB) and employ techniques to enhance the trustworthiness of our work. The analysis was data rather than theoretically driven; we held the viewpoint that the language used by the participants reflected their meaning of their experiences of START.

The seven steps of template analysis [17] were applied across the data sets of the two different participant groups. This involved initially the analysis of the interviews with the teachers as one data set and the focus groups as a different set. The first phase involved familiarisation with the data. Both researchers read and re-read the transcripts of the interviews and focus groups. For the second phase of preliminary coding, both researchers highlighted the relevant units of information they believed contributed to understanding the participant’s perceptions and experiences of the START initiative. The third phase, clustering, involved organising the codes into groups (clusters) of meaningful broad and narrow themes. The two researchers employed the initial three steps independently on four of the teachers’ interviews and four focus group interviews then carried out the fourth phase together. Using Word and Excel documents and flip charts, initial versions of the coding templates, consisting of the broad and narrow themes, were generated. The templates were populated with excerpts of raw data from the different sources. This process helped us to refine further and agree the templates and to ensure that the meaning of each theme was explicit. In the fifth step, each researcher applied the coding templates to the interviews and focus groups they had not yet reviewed. This iterative process allowed for modifications and development of the initial version of the coding templates. We carried out this sixth step together; looking at both templates it was apparent that the themes were common across the data sets of interviews and of focus groups. We combined these into one template and developed the version of the template that was then applied across the full data set. Adding relevant field notes to the template document provided an insight into the context of the findings (e.g. a finding that the children recalled with excited voices). Step seven of template analysis [17] informed the reported findings and discussion within this paper.

Analysing the data independently and then coming together for some of the phases meant that the researchers had to reflect on and verbalise their decisions, discuss the similarities and any differences in relation to their analysis and provide a rationale for the themes included in all reiterations of the templates. We presented the findings to the other members of the team and addressed any points of feedback. This process of *independent scrutiny* [17] and reflexivity contributed to the decision trail (Audit Trail [20]) and the rigour of our study.

## Results

We report the findings using descriptive summaries supported with examples of direct quotes from the data [21]. Data were grouped into four broad themes.

1. START—it’s relevant across the primary school curriculum
2. It was doing science not just reading about it
3. We created our trial
4. Improving START

### START—it’s relevant across the primary school curriculum

All participants were asked why they decided to participate in START. START interested teachers because they perceived that it provided a new vehicle to promote science within their classroom.

Teachers and their students highlighted that the personal interest of the teacher in *doing science* (the term was used in its broadest form) influenced their decision to engage with START. Some of the teachers referenced their previous education and/or employment within scientific fields, highlighting that this competition not only appealed to their personal interests but also enabled them to draw on their specific skill set.

*... because of my scientific background …I was doing my own science experiments in class anyway and it’s* [START] *just an extra adventure* (Teacher 08)

*I had just an interest in science …. I am involved with planning* xxx [named scientific event] *so I’d be very interested in science and it’s something I really try to promote here since I came* (Teacher 01)

The teachers suggested that their interest in and knowledge of science meant that they saw what START could bring to their classroom. Some suggested the START initiative provided a new framework on which they could introduce and advance aspects of the science requirements of the primary school curriculum.

*It’s something that we always try to focus on when are doing science—is fair trials and what a fair trial is, so it fits very nicely with that, so we just felt we could do a full unit of work on that and it fitted in nicely with that* [science] *… that’s what attracted me to it* (Teacher 01)

*It gave me another way to look at it* [science] *and another way to think about it. Because I’m always adding to my little portfolio of science* (Teacher 08)

The interest the teacher had in scientific subjects was not lost on the children and they saw how the teacher’s interest was linked to the class’s exposure to START.

*Well, Teacher x* [teachers name] *does A LOT of STEM subjects* … x [mentioned teachers name*] is always getting us to do things like this* (Focus Group G)

*Yea, its x’s* [teachers name] *kinda thing* (Focus Group B)

The teachers spoke of the uniqueness of the START competition, stating that it was unlike the more regular competitions their school participates in (e.g. mainly sporting activities, colouring and story writing competitions). In addition to the science portfolio, some of the teachers saw the requirements of the START initiative as having the potential to draw from numerous aspects of the primary school curriculum (e.g. numeracy, literacy). START had the potential to build on skills (e.g. IT skills) the children had developed during the previous semesters.

*I thought it would be good for them, for critical thinking and thinking outside the box, and coming up with their own ideas* (Teacher 03)

When referring to the experiences of participating in START, teachers and children noted that the learning was not limited to science or to trial methodology. The children and teachers identified the benefits of group work and the skills of communication and cooperation that were required to support a successful project. The teachers spoke extensively of the contribution of START to “*benefits across the curriculum*”, and to “*transferrable skills*”.

*You know a broad range* [of skills] *from literacy, to numeracy, to science, to everything* (Teacher 07)

*we are able to link this with their English or literacy programme as well for oral language and to question those type of thing so it was helpful in that we were able to link it to a lot of subjects actually so that was good* (Teacher 01)

The teachers listed how START had contributed to literacy and maths, advanced the children’s engagement with technology and touched on areas of their social, personal and health education. Examples offered by the teachers indicated that the children had to engage in cross-age peer learning, with more senior and indeed more junior classes, and have the tools to describe their study to all ages of children. Some children presented their project to their school to encourage recruitment, others presented regular updates in relation to study progress and the findings of their work, some interviewed the teachers and their peers on aspects of trial processes (some even embedded Studies Within a Trial (a SWAT [22]) in their projects), while others made videos to showcase their work to the judges of the competition. From the gathering of their ideas in a scrapbook to recording an online diary, each group found their way to verbally map how the START initiative contributed to the learning outcomes of the primary school curriculum.

*It can work across the whole curriculum … we weren’t just doing a science competition and we weren’t just doing technology. They developed literacy skills. They developed oral language skills. There was interviewing. There was, you know they were creating rather than just merely working through a worksheet, or a book. …. it gave them great scope to investigate … the science curriculum is full of investigating and developing skills, of a scientist. And sometimes that is lost, you know. Even the movie making you know, there was drama. Because they had to act out the little video … they scored up all of the answer sheets. They calculated the average score, they did percentages … and using Google sheets to draw up an Excel sheet. And make a bar graph, you know all those things. It’s maths, it’s everything you know* (Teacher 07)

Some of the children touched on these points and were aware that their teacher was using the START competition as a learning activity for many subjects. These comments were offered with humour and a suggestion that they would be more aware the next time.

*If we started again I think like* [teachers name] *would have to be like sneaky about it and be like, like incorporate it into English and be like now what ideas would you have for a trial and like you’d be, get us to answer questions on trials in English and then she’d have to, and then be like ‘oh this is for this trial’* (Focus Group A)

The teachers recalled how the START initiative spoke to their need to introduce science to the classroom. START encouraged activities that contributed to other areas of the primary school curriculum and so was held as valuable in this regard. Children offered some explanations as to why the teachers introduced START to their classes; however, they did not see these benefits until they engaged with the process.

### It was doing science not just reading about it

The children identified how they felt START differed from many of their learning activities, especially they way they learned about science. The most exited recall related to their experiences of *doing* their project; the children, across all focus groups, made reference to the fact that they had learned from doing.

*In our text books it just tells you something and then makes you do some questions on it, whereas in this* [START] *it made you look at something and keep looking at it and then look back at it again* (Focus Group E)

The initial enthusiasm for START was not held by all of the children; some said they did not get a choice and were just told that their class was going to enter the competition. Participants within two focus groups noted they were not too excited initially, fearing that this was just homework by another name.

*At first we were all really excited and then we found out how much work it took, so then we kind of got less excited but then when we were actually doing it, then it was way better than we thought it would be* (Focus Group F)

However, even the children initially fearing that it was “*just homework in disguise*” were “*won over once we got into it*”. This new way of engaging with science, as the children saw it, was held as an important aspect of their experiences.

Reference was made to the traditional way in which they learned about science. This usually involved reading—reading about an activity, reading about the specific way to do something and having someone recount to them exactly what should be done and how. Their experiences of participating in the START initiative were viewed as being different to their normal class work; they were not reading about someone else’s work in retrospect but rather they did and lived the work, working it out as they went through the process of the trial.

*It’s different because in the science book it’s telling you about it, but when we were doing the project, we were trying to figure it out for ourselves* (Focus Group A)

The children spoke of their experiences of brainstorming—asking questions, problem-solving, finding answers. These techniques were used across all steps of the trial process*.* The teachers also commented on the benefits of this approach to learning and compared it to the rote learning techniques that informed their own schooling and indeed some of the other teaching strategies that they used.

*when we were in school, you know you just, you didn’t research as such, you didn’t investigate, you just read about everybody else’s. And had to regurgitate it. … like anybody can read a book but it’s lovely to go off and find out for yourself and not be restricted by a book* (Teacher 07)

*They are learning a huge amount from that* [START] *and they’re probably benefiting more from that. They will, long after they’ve gone from this school, they will forget the things that I had as learning objectives from a text book or from a PowerPoint or whatever. They will remember START. I think it is something that will stick with them throughout* (Teacher 06)

Some of the classes had similar experiences of experiential learning with other school projects they had undertaken (e.g. technology projects); others suggested that this was a new way of learning about science. The children marvelled at their learning, suggesting that it occurred almost unbeknownst to them; it was not schoolwork. This ‘was fun’.

Child: *You don’t actually think of it as work, it didn’t just pop into your head when you were correcting work or like playing games …. You were like – ‘oh, this is work, I’m working for this’! It’s just like, you are kinda having fun at the same time.*

Child: *Just like …‘this is awesome, this is so fun’ and we have time off work, hooray!* (Focus Group D)

Child: *And when it’s a book you just, it’s just there in front of you and you just find out straight away but when you’re doing a project it’s fun …*

Child: *you have to dig deeper and you have to find it out a different way* (Focus Group A)

The teachers recognised also that the children viewed this as different to their normal learning experiences.

*they were kind of going oh this is different, this is interesting. This is games, we are not going to be doing work, we are not working here. We are just doing games. And even putting them in charge of giving the tests and correcting the tests and putting the scores together like they didn’t see it as work. They just saw it as something different and enjoyable. Whereas you know you are going ‘it is work’, because you are correcting maths you are doing maths!* (Teacher 05)

Across the focus groups the children said that the START initiative brought something new to the classroom. The children suggested that START was different to competitions they had engaged with in the past; they went as far as to say that it was different to their school work and this is what made it attractive to them. This *newness* was considered exciting, something worth engaging with, something that enabled release from what the children considered to be *normal* work and the normal way they engaged with science. The benefits associated with this learning strategy were also acknowledged by the teachers.

### We created our trial

The children went to great lengths to describe the intricacies of their trials. All children recounted that the START competition provided their first exposure to trial processes; however, the complexities of trial methodology were not lost on them. The classes and teachers spoke at length of “*how they got their head around it*”, noting that an understanding of certain concepts was required to engage with the trial process. This understanding often emerged as a result of the children’s engagement and commitment to the project. Regardless of the order of learning, the children and the teachers were proud that the children were able to understand and engage to the extent that they planned, conducted, analysed and reported a trial.

The children spoke at length of their accomplishment and conducting their trial was a badge of honour. “*We did it, we did it all*”, “*it was our trial, our idea, our work*”.

Child: *Teacher* [names teacher] *kind of directed us towards the right direction and then we decided what we wanted*

Child: *We took it from there* (Focus Group D)

*… we got to take control of everything* (Focus Group B)

The teachers agreed and spoke of their role in facilitating learning—providing support, yet encouraging independence, ensuring that the children had ownership of the trial noting “*it was their work*”, “*they had to do everything*”*.* The teachers spoke at length of ‘critical thinking’ skills and provided many examples to demonstrate the way the children “*found the answers to problems*”, something that they said was not their usual practice.

The children spent a considerable length of time, in all the focus groups, talking about and highlighting their understanding of all stages of their trial. They made reference to their question, how they came up with it, if it was something they could test, would it provide a fair comparison. This seemed to be the stage of the project that caused the greatest anxiety for most of the schools; the children spoke of the need to get this right, to ask a proper question, to have a “*fair comparison*”. They all provided a detailed rationale for their chosen topics and highlighted some of the reasons for discarding other choices.

*We did groups and everyone, we did like a Dragons Den kind of thing where everyone presented their ideas* [about the question] (Focus Group C)

*We kind of tried to pick a thing that we could do within the classroom rather than like having to go somewhere, that we would do it here* (Focus Group A)

Some of the schools used online packages to prioritise the questions that were proposed; others chose a pragmatic approach of settling on a question that was considered easy to explore within the resources available to their school.

*We did lots of brainstorming and putting them in pairs and getting them to come up with ideas and then putting them in groups to come up with more ideas. Kind of worked on what we could do in the timeframe, what we could test? What would be measurable? And then just, I think we voted on it in the end what we’d do ….. It took a few days, now it wasn’t quick. Because they might come up with a bit of an idea and then getting them to fill in the rest of it* (Teacher 05)

*So to actually figure out what the test was going to be. That might’ve been the hardest part; you know to distil down through all the ideas. And figure out, well which one can we actually test, you know. That could’ve been the hardest part of it* (Teacher 08)

The children spoke of this phase with some impatience. They wanted to get started and not spend so much time talking about their question. However, it became apparent as the interviews progressed that the children were not just talking about identifying the question of their trial but also what outcomes they would measure and what methods they would use to gather and analyse their data. Some identified that, with hindsight, they now saw the importance of this preparation and how it helped them once they started their trial.

*I think coming up with the idea might’ve been* [the worst part of the experience]. *Because like you had to think of loads of ideas. But then when we had the idea it was easier and more enjoyable* (Focus Group B)

All made reference to the *groups* in their trial. The participants of some trials did not know if they were allocated to the intervention or control group and other participants were not aware of the outcomes being measured; all classes provided a rationale as to why they thought such blinding was necessary and how it might contribute to their trial (the ‘secrecy’ also added to the fun the children had with the project).

Child: *We were told not to tell before the test of what we were doing. So you know*

Child: *In case you got skewed results*

Child: *Word might go the class* (Focus Group F)

*Something they found really, really fun was keeping the secret from the rest of the school, doing that, so you know that, so that the children that who were being tested, so it wouldn’t compromise their trial, so having that responsibility as scientist to, you know, manage their trial was really good. They really enjoyed that* (Teacher 08)

Lengthy stories were offered in relation to measurements, testing, “*getting our results*”. We refer in our field notes to the excitement and enthusiasm the children displayed during these elements of recall (speaking over one another, becoming more animated). They described how they waited anxiously for the findings of their trial, how they really wanted to know the answer to their question, they wanted to tell other people the answer to their question. While the children did not, for the most part, use the language most typically used by trialists in relation to this phase of their projects, they were able to explain what they did, why they did it and what they had to do with the information they gained.

Child: *We explained our trial* [to other classes] *what we are doing*

Child: *We explained like so, what we were doing, why we were doing it*

Child: [we said] *we were going to like, we would split them up into groups but we wouldn’t tell them like what the groups were or how we were going to split them up. And they would x* [referred to the intervention] *each day every day for two weeks I think it was* (Focus Group D)

How they presented their trial (to their school, for the competition of START) varied in the schools. Some groups drew on methods they had used in the past for other class work. Some used this as an opportunity to engage new skills and technologies (e.g. scrap books, online presentations, podcasts, written reports, photos). Regardless of the methods employed, all children identified an awareness that they needed to ensure that the findings of their work were presented in an understandable manner.

All the children said they would enter START again because they now knew how to conduct a trial. Some said that they would re-do their original trial and would refine it as a result of learning during the process. For others, they would do something completely different. Regardless of whether the children said they would identify a new question or re-visit their old one, all suggested re-entering START because now they would know what to do, they knew what was involved and they would be “*quicker*” at doing everything the second time.

*We’d get into groups and pick the question; we’d be quicker ‘cause we know now what has to be done* (Focus Group G)

All groups talked through each phase of their trial and while some groups went back over points or children interrupted each other, the children provided an explanation of what they did in their trial. They did not (aside from a few examples) use the language of trial methodologists; however, they were able to talk about their work in a way that highlighted an awareness of the basic concepts.

### Improving START

This theme highlights clearly the limitations of the initiative as experienced by these participants and their suggestions to help inform further iterations of START. When describing their opinions in relation to START the teachers felt it necessary for teachers to have access to more resources to help them understand the trial processes and the sequence of steps therein. The children also had opinions in relation to additional resources. Both children and teachers suggested that an acknowledgement of participation should be provided to all schools and not just those ranked as the top three in the competition.

The teachers and children spoke of the learning that occurred during the children’s engagement with START. The teachers highlighted that for the children to learn, the adults had to have an ability to explain the concepts in a clear and simple manner. Some of the teachers found this challenging and suggested that they would have benefited from additional resources.

*More information on the ‘what is a trial?’ and you know even a link to just something that would explain it to both teachers and pupils so it’s clear from the beginning you know so I had, you had to kind of go and look. Do a bit of research yourself to try and find like videos and that. Or more pupil-friendly, so that was the thing about it, I didn’t find a lot of information. I had to go kind of looking myself for resources, so I think you probably get more people involved if there was a bit more on the website about trials* (Teacher 04)

Some of the teachers suggested that more guidelines with explicit timelines for each phase of the trial process would be useful and would have reassured them in relation to the length of time spent on each stage.

Limited child-friendly resources were also raised as an issue. Children and teachers offered suggestions as to what would be beneficial for the age group involved.

*get the winners to make a long video* (Focus Group B)

*a video for it … tells them and it’s in a child-friendly way but it is very specific, it is very driven it’s, you are under no illusion at the end of it what is wanted* (Teacher 02)

Comments were made in relation to a more interactive platform to support website resources and enable the schools to engage with more frequent, staggered reports, at different stages of the trial and not just their final submission.

*Put in a little resource that, like a sketchpad or a key-note or something that they could put up the headings for the kids on the different topics of discussion or whatever your next step is. Step one, step two, step three, you know, and just be like pages even for step three, sketch your ideas here and let them write on it or, you know, I just thought that was handy, we just made our own kind of to keep on track* (Teacher 06)

As well as online resources, teachers suggested that they would have liked more support from a researcher. Some of the participants, children and teachers alike, used the title *Start Ambassador* and proposed that someone from the START team should be linked to the participating schools. While someone was available to the schools, and their contact details were listed on the START website, the teachers and children felt that this needed to be a more formal arrangement and that this ambassador could visit the schools during the phases of their trial. They described this role as “*a link between university and us*”, a person to explain the concepts, guide the project timelines, answer questions when needed but yet remain outside the running of the trial.

*If we told you guys what we were going to do and you look it up and just explain a couple of stuff to us, like to make it easier before we start, then we could say ‘oh that’s what it is!’* (Focus Group E)

*Our focus is getting the project together, whereas if we had someone to explain it well it would help us and the kids* (Teacher 08)

Recognition of the work done by all the entrants of START was something teachers and children raised. The children and teachers suggested that all schools should receive some acknowledgement of their participation regardless of ranking, something that they could keep on display in the school (all schools received a certificate of participation). This would allow them to show-off to the other classes, their parents and visitors to the school.

*if everybody was to get a plaque every year and then just the winners were brought in* [to NUI Galway] *and said, like you know, still there was a sheet sent out to say ‘fair play. There’s your plaque. Huge achievement’.* (Teacher 02)

*A trophy to put up in the school* (Focus Group G)

## Discussion

START is the first initiative in Ireland, and possibly internationally, that encourages primary school children to design, conduct, analyse and report a trial within the context of a competition. The findings of this study provide a more in-depth picture of the strengths of START and the benefits of engaging with the initiative from the perspective of participant children and their teachers. The weaknesses have also been highlighted by the participants of this study, as have areas that need to be considered and changed for future competitions.

Teachers and children alike identified START as having the potential to contribute substantially to key aspects of the Irish primary school curriculum, not just the children’s understanding of trial processes. The Irish Primary School Curriculum [23] identifies that scientific investigation, for 10–12 year olds, must be employed to develop science skills and also children’s abilities to generate solutions to practical problems. The curriculum stipulates that children should be able to: i) question; ii) observe; iii) predict; iv) investigate and experiment; v) estimate and measure; vi) analyse; vii) record and communicate; viii) evaluate. The teachers indicated that START contributes to these curricular requirements of science within the classroom. The children and teachers noted that a trial, like any scientific experiment, requires participants to adhere to a defined set of rules and procedures to get the best results.

The teachers valued that the initiative could also encourage activities that contributed to learning in maths, languages and literacy, visual arts, information and communication and social, personal and health education (subject areas identified in the primary school curriculum). The process evaluation of the IHC project notes that the intervention to teach primary school children to assess claims about treatment effects was not incorporated into the curriculum; rather, it was in additional to it [16]. This was held as something that could be a burden on the teachers and an impeding factor. A curricular mapping of START to the learning objectives of the Irish primary school curriculum could be useful to ensure that participation is not seen as a burden but as a tool to help deliver national requirements.

As well as contributing to scientific literacy and other subject requirements of the primary school curriculum, participating in START led children to identify and learn about the way they learn. Phrases such as ‘*doing science not just reading about it*” and “*it wasn’t learning, it was fun*” demonstrate how the children differentiated between this and what they referred to as their *normal* learning experiences. As noted, START draws on the Theory of Experiential Learning [2, 3], a philosophy of experiential learning that focuses on “a logical sequence which involves perceiving a problem followed by its articulation, the formation of a hypothesis for finding a solution, experimentation to test the hypothesis and finally giving reflective consideration to the consequences for society” [3] (p. 326). This mirrors the steps of learning described by the participants in this study. The children had the support of both their teachers and the START resources; however, their personal involvement in their learning seemed to contribute to their ownership of the trial—it was their work, their project and their learning.

The children participating in this study designed, conducted, analysed and reported their own trials. This study demonstrates the importance attached to engaging with and completing all the steps of the trial process. The findings of this study tell clearly of children’s ability to talk about their experiences and in doing so demonstrate their awareness of trial processes. We do not know if increasing public awareness and understanding of randomised trials increases future participation in trials, but it seems reasonable to suggest that this is a necessary precursor to ensuring adequate recruitment to clinical research trials [24].

Participants in this study identified aspects of START that require attention. A key finding relates to the resources supporting the participants of START. Teachers suggested that they would have benefited from more guidance on trial methodology with the view that they could then provide more support to the children. Evidence suggests that students can achieve more by working in small groups than working on their own [25]; however, this is only achieved when teachers can provide adequate instruction and know when to intervene and when to stand back, a concept termed ‘scaffolding’ [26]. Teachers cannot make these decisions if they do not have confidence in their own knowledge and understanding in relation to conducting a trial. Developing additional support for teachers (and for children) and providing them with different levels of scaffolding, as required, may address this need.

While the teachers and children did not raise concerns about the competition aspect associated with this initiative, participants of this study drew attention to recognizing the effort of all participants of START and not only those schools who were shortlisted for the START award.

The findings of this study may have wider interest outside those involved in developing and improving START. The focus on trial methodology and the discourse in relation to waste in trials [27], the establishment of centres and networks such as Trial Forge in Aberdeen (www.trialforge.org), the HRB-Trials Methodology Research Network in Ireland (www.hrb-tmrn.ie) and the MRC-NIHR-Trials Methodology Research Partnership (https://www.methodologyhubs.mrc.ac.uk/about/tmrp/), speak to the challenges trialists currently face. It is widely accepted across the trial community that taking part in trials can be beneficial for healthcare, but a lack of public understanding around trials may limit overall participation in trials [28–30]. Educating young children about the importance of trials may help contribute to a broader awareness, understanding and engagement with trials.

### Limitations

While there is no set rule to establish the most appropriate sample size in qualitative research [31], it may be critiqued that this study is informed by eight schools only. However, the value of conducting interviews with eight teachers and focus groups with 61 children cannot be ignored; adequate data were obtained to meet the objectives of this study [32].

In some cases, teachers identified the participants for the focus groups and we recognise that children may have been picked for certain reasons that we were not aware of and other children in the class may have provided a different viewpoint.

All of the participants were aware of the research team’s involvement with the START initiative. Some of the participants had interactions with the team in relation to START before the interviews. While the interview guides informed the episodes of data collection, participants may have been more complementary about START because of their knowledge of the contribution the researchers made to the initiative. The research team are also aware that their involvement with the START initiative may have influenced the manner in which they conducted this study and viewed and interpreted the data. We have endeavoured to be reflexive when reporting this study.

## Conclusions

The participants of this study have not only highlighted the benefits of participating in the START competition but have also clearly indicated steps we can take to ensure that the support and resources available to future schools are developed appropriately. Particular areas of focus lie in the information available on the website and developing even closer links with the START team. Work is currently underway to address the recommendations for more resources noted by the children and teachers.

While recalled as innovative and fun, the findings of this study demonstrate the value of the START initiative. The children were able to identify and engage with the stages of trial processes. While they may not have used the language of trialists, they were aware of and able to address each stage, highlighting the relevance of all methods they employed (or those they did not but would in subsequent iterations). START seeks to raise awareness of randomised trials so that these children can better engage with them as future researchers, patients and public citizens.

## Supplementary information

**Additional file 1.** Standard for Reporting Qualitative Research (SRQR).

## Data Availability

The datasets used and/or analysed during the current study are available from the corresponding author on reasonable request.

## Abbreviations

HRB: Health Research Board
HRB-TMRN: Health Research Board-Trials Methodology Research Network
IHC: Informed Health Choices
MRC: Medical Research Council
NIHR: National Institute for Health Research
SRQR: Standards for Reporting Qualitative Research
START: Schools Teaching Awareness of Randomised Trials
SWATS: Studies within a trial
